# Asociación entre calidad y cantidad de sueño con índice de masa corporal en adolescentes universitarios: Estudio transversal*

**DOI:** 10.15649/cuidarte.3032

**Published:** 2023-12-21

**Authors:** Beatriz del Ángel Pérez, Ricardo Lara Pérez, Paulina Aguilera Pérez, María de los Ángeles Fang Huerta, Florabel Flores Barrios

**Affiliations:** 1 . Universidad Autónoma de Tamaulipas, Tampico, México. E-mail: bdelange@docentes.uat.edu.mx Universidad Autónoma de Tamaulipas Universidad Autónoma de Tamaulipas Tampico Mexico bdelange@docentes.uat.edu.mx; 2 . Universidad Autónoma de Tamaulipas, Tampico, México. E-mail: drlpr@hotmail.com Universidad Autónoma de Tamaulipas Universidad Autónoma de Tamaulipas Tampico Mexico drlpr@hotmail.com; 3 . Universidad Autónoma de Tamaulipas, Tampico, México. E-mail: paguiler@docentes.uat.edu.mx Universidad Autónoma de Tamaulipas Universidad Autónoma de Tamaulipas Tampico Mexico paguiler@docentes.uat.edu.mx; 4 . Universidad Autónoma de Tamaulipas, Tampico, México. E-mail: mfang@docentes.uat.edu.mx Universidad Autónoma de Tamaulipas Universidad Autónoma de Tamaulipas Tampico Mexico mfang@docentes.uat.edu.mx; 5 . Universidad Autónoma de Tamaulipas, Tampico, México. E-mail: fflores@docentes.uat.edu.mx Universidad Autónoma de Tamaulipas Universidad Autónoma de Tamaulipas Tampico Mexico fflores@docentes.uat.edu.mx

**Keywords:** Sueño, Sobrepeso, Obesidad, Adolescente, Estudiantes de Enfermería, Sleep, Overweight, Obesity, Adolescent, Students, Nursing, Sono, Sobrepeso, Obesidade, Adolescente, Estudantes de Enfermagem

## Abstract

**Introducción:**

La modificación en los patrones de sueño incrementa la susceptibilidad para la ganancia de peso.

**Objetivo:**

Estimar la asociación entre la calidad y la cantidad de horas de sueño por noche con el Índice de Masa Corporal (IMC) en adolescentes universitarios mexicanos.

**Materiales y métodos:**

Estudio transversal en estudiantes de nuevo ingreso de Enfermería en una Universidad Pública de México. Muestreo por conveniencia de n=134 estudiantes (18-19 años), voluntarios y matriculados en la facultad. La calidad y cantidad de sueño se midió con el Índice de Calidad de Sueño de Pittsburgh. El IMC se calculó con la fórmula estándar. El protocolo fue aprobado por el Comité de Ética de la facultad, los participantes firmaron el Consentimiento Informado.

**Resultados:**

Edad promedio de 18,21 años (DE= ,42 años), 82,84% mujeres, 45,52% (IC 95%= 35,46 - 52,86%) con sobrepeso u obesidad. El 44,02% de los participantes reportó dormir < 6 horas por noche, 50,00% refirió una mala calidad de sueño en las últimas cuatro semanas. Después de ajustar por edad, sexo y ocupación, la calidad del sueño no se asoció con el IMC (OR= 1,01; IC 95%= ,94 - 1,08; z= ,31; p= ,75); el incremento en el IMC se asoció con una mayor probabilidad de dormir 6 horas o menos (OR= 1,08; IC 95%= 1,01 - 1,16; z= 2,25; p= ,02).

**Discusión:**

Las alteraciones hormonales, los factores conductuales y el entorno pudieran explicar la asociación.

**Conclusiones:**

Es recomendable implementar estrategias educativas para mejorar la higiene del sueño en esta población.

## Abstract (en)

**Introduction:**

Changes in sleep patterns increase susceptibility to weight gain.

**Objective:**

To estimate the association between the quality and quantity of sleep hours per night and Body Mass Index (BMI) in Mexican college adolescents.

**Materials and methods:**

Cross-sectional study conducted among incoming nursing students in a public university in Mexico. Convenience sampling was used, involving n=134 students (18-19 years old), volunteers, and enrolled in the nursing program. Sleep quality and quantity were measured through the Pittsburgh Sleep Quality Index (PSQI). BMI was calculated using the standard formula. The protocol was approved by the School of Nursing Ethics Committee, and the participants signed informed consent.

**Results:**

Mean age 18.21 years (SD= .42 years), 82.84% female, 45.52% overweight or obese (95% CI= 35.46 -52.86%). 44.02% of participants reported sleeping < 6 hours per night, and 50.00% reported poor sleep quality in the past four weeks. After adjustment for age, sex, and occupation, sleep quality was not associated with BMI (OR= 1.01; 95% CI= .94 -1.08; z= .31; p= .75); increased BMI was associated with a greater likelihood of sleeping 6 hours or less (OR= 1.08; 95% CI= 1.01 - 1.16; z= 2.25; p= .02).

**Discussion:**

Hormonal changes, behavioral factors, and the environment may explain the association.

**Conclusions:**

It is advisable to implement educational strategies to improve sleep hygiene among this population.

## Resumo

**Introdujo:**

A modificado nos padróes de sono aumenta a suscetibilidade ao ganho de peso.

**Objetivo:**

Estimar a associagáo entre a qualidade e a quantidade de sono por noite e o índice de massa corporal (IMC) em adolescentes universitários mexicanos.

**Materiais e métodos:**

Estudo transversal de estudantes de enfermagem ingressantes em uma universidade pública no México. Amostragem por conveniencia de n=134 estudantes (18-19 anos de idade), voluntários e matriculados na faculdade. A qualidade e a quantidade do sono foram medidas com o Índice de Qualidade do Sono de Pittsburgh. O IMC foi calculado com a fórmula padráo. O protocolo foi aprovado pelo Comite de Ética da faculdade e os participantes assinaram o Termo de Consentimento Livre e Esclarecido.

**Resultados:**

Idade média de 18,21 anos (DP= ,42 anos), 82,84% do sexo feminino, 45,52% (IC 95%= 35,46 - 52,86%) com sobrepeso ou obesidade. 44,02% dos participantes relataram dormir < 6 horas por noite, 50,00% relataram qualidade de sono ruim nas últimas quatro semanas. Após o ajuste para idade, sexo e ocupado, a qualidade do sono náo foi associada ao IMC (OR= 1,01; IC 95%= ,94 - 1,08; z= ,31; p= ,75); o aumento do IMC foi associado a uma maior probabilidade de dormir 6 horas ou menos (OR= 1,08; IC 95%= 1,01 - 1,16; z= 2,25; p= ,02).

**Discussao:**

Alteragóes hormonais, fatores comportamentais e o ambiente podem explicar a associagáo.

**Conclusóes:**

É aconselhável implementar estratégias educacionais para melhorar a higiene do sono nessa populagáo.

## Introducción

Tanto la obesidad como el sobrepeso se han convertido en una epidemia^1^, en México, afectan a más de una tercera parte de la población adolescente (38,5%), situación que posiciona al país como el de mayor prevalencia^2^^,^^3^. Al exceso de peso se le atribuye el desarrollo de otras enfermedades crónico- degenerativas y el deterioro de la salud mental, lo que repercute negativamente en indicadores escolares, sociales y económicos^4^.

Distintos factores se vinculan con el desarrollo de la obesidad, principalmente la dieta y la actividad física, sin embargo, desde hace una década también se ha estudiado la implicación de una mala calidad de sueño con el sobrepeso y la obesidad^5^^,^^6^^,^^7^, situación que afecta de forma particular a los estudiantes universitarios. En cuanto al ámbito académico, los estudiantes realizan una serie de modificaciones en su estilo de vida y hábitos de sueño, derivado de los nuevos retos estresantes de la educación superior, convirtiéndolos en individuos con un mayor riesgo para la ganancia de peso^8^^,^^9^^,^^10^^,^^11^^,^^12^.

La mala calidad de sueño afecta entre el 12-40% de la población a nivel mundial^13^^,^^14^. En estudiantes universitarios, su prevalencia se ha estimado del 36,5% en hombres y de 39,1% en mujeres^11^. En general, las mujeres reportan mayor necesidad de sueño y una menor sensación de descanso en comparación con los hombres^5^^,^^11^. Asimismo, los estudiantes universitarios duermen en promedio menos de siete horas por noche^10^^,^^12^.

Autores han reportado una asociación, principalmente negativa, entre la calidad y la duración del sueño con el Índice de Masa Corporal (IMC) o con el porcentaje de grasa corporal^7^^,^^11^^,^^12^^,^^15^. También se ha reportado que la calidad de sueño no se asocia con el IMC, sino que intervienen otros factores como la actividad física^9^^,^^10^^,^^12^, la utilización de dispositivos electrónicos y la alimentación^9^^,^^16^.

La asociación entre la calidad del sueño y el sobrepeso u obesidad en población universitaria no ha sido del todo clarificada. Por una parte, se ha reportado que el IMC se asocia mayormente con la cantidad de horas de sueño que con la calidad del sueño^12^^,^^15^^,^^17^. Además, la población de estudio se ha centrado en adolescentes entre 9-17 años. Por tal motivo, el objetivo del estudio fue estimar la asociación entre la calidad y la cantidad de horas de sueño por noche con el IMC en adolescentes universitarios mexicanos.

## Materiales y Métodos

### Población

Como parte del Programa de Fomento a la Salud implementado en el 2012 por la facultad de Enfermería todos los estudiantes de nuevo ingreso son informados, capacitados y valorados en características relacionadas con la nutrición, la actividad física, el manejo del estrés y la prevención de adicciones en el Módulo de Salud de la facultad. Derivado de este Programa, se realizó un estudio transversal durante el mes de marzo de 2018 en estudiantes universitarios (n= 175) de nuevo ingreso a la Lic. en Enfermería de una Universidad Pública en Tamaulipas, México.

### Muestra y muestreo

De los estudiantes de nuevo ingreso, se obtuvo una muestra no probabilística por conveniencia de n= 134 estudiantes cuyos criterios de inclusión fueron todos aquellos participantes voluntarios y matriculados en la facultad; los criterios de exclusión fueron la presencia de embarazo o ser menores de edad (<18 años), esto debido a la dificultad para conseguir el asentimiento informado. Los criterios de eliminación fueron cuestionarios con registros de datos con valores extremos en la cantidad de horas de sueño por noche (<4 horas o >12 horas)^18^ y aquellos que proporcionaron información incompleta en el autorreporte, por tanto, no se consideraron n= 41 estudiantes en el presente estudio.

### Procedimiento e instrumentos para la recolección de información

Para la colecta de información se aplicó un instrumento, durante la valoración de ingreso, integrado por tres apartados. El primero de ellos, constituido por las características sociodemográficas como la edad, el sexo, la ocupación (estudiante o estudiante y trabajador) y el estado civil (soltero o casado). El segundo, compuesto por las medidas antropométricas: peso (kg), talla (m) e IMC. Para la medición del peso y la talla se utilizó una báscula con estadímetro marca BAME modelo 420, siguiendo procedimientos estandarizados acorde a la Norma Oficial Mexicana NOM- 047- SSA2- 2015, para la atención a la salud del grupo etario de 10 a 19 años. Se les solicitó a los estudiantes que se retiraran los zapatos antes de subirse a la báscula, vestir lo más ligero posible, colocarse de espalda al estadímetro y distribuyendo su peso en ambas piernas. La estatura se midió con el estadímetro de la misma báscula, pidiéndoles mantener una postura erguida. La variable del IMC se calculó mediante la fórmula estándar: peso(kg) / talla(m)2, posteriormente fue categorizada considerando la edad y el sexo de los participantes, acorde a las recomendaciones de la Organización Mundial de la Salud para población de 5 a 19 años^19^. El último apartado del instrumento incluyó la valoración de la calidad del sueño y cantidad de horas de sueño.

La calidad del sueño en las últimas cuatro semanas al momento del estudio se midió con el Índice de Calidad de Sueño de Pittsburgh (PSQI, por sus siglas en inglés). Es un instrumento que consta de 19 ítems agrupados en 7 componentes: calidad subjetiva de sueño, latencia de sueño, duración de sueño por día, eficiencia habitual de sueño, trastornos del sueño, medicación hipnótica y disfunción diurna. Estos componentes se puntúan en una escala de 0-3 puntos, posteriormente se suman los puntos de cada componente para generar un puntaje total que oscila entre 0-21 puntos, valores más altos indican una menor calidad de sueño. Además, este puntaje puede ser dicotomizado en 5 puntos, de 0-5 puntos significa una buena calidad de sueño, mientras que un puntaje de 6-21 puntos indica una mala calidad de sueño^11^. La confiabilidad del instrumento es aceptable (a de Cronbach > 0,70) tanto en la versión original en inglés^20^ como en su adaptación al español en población mexicana^21^, en el presente estudio fue de 0,74.

La cantidad de horas de sueño por noche se midió a través de la pregunta del PSQI: “En las últimas cuatro semanas, en promedio, ¿cuántas horas ha dormido por noche?”, y clasificada en base a si cumplían (> 6 horas) o no cumplían (< 6 horas) las horas recomendadas de sueño diarias conforme lo establecido por la Fundación Nacional del Sueño^22^.

### Análisis Estadístico

En el análisis descriptivo se presentaron los atributos de los participantes como frecuencias y porcentajes, media (desviación estándar, DE) o mediana (Q1 - Q3), según fuera apropiado. Las variables sociodemográficas y de salud se compararon por grupos de calidad de sueño y cantidad de horas de sueño usando pruebas tipo Ji- Cuadrada de independencia, exacta de Fisher, t de Student o la prueba no paramétrica U de Mann- Whitney, dependiendo de la naturaleza y características de las variables.

Las pruebas estadísticas utilizadas se fundamentaron en lo siguiente: En las tablas de contingencia de dos vías, cuando se observaron celdas con valores esperados <5, se utilizó la prueba exacta de Fisher para valorar asociaciones simples o la prueba Ji- Cuadrada de Independencia en caso contrario (celdas con valores esperados >5). Para valorar diferencias en las medias de las variables numéricas por grupos de calidad y cantidad de sueño en su forma dicotómica se utilizó la prueba t de Student en aquellas variables donde no se rechazó la prueba de hipótesis de normalidad (edad y cantidad de sueño, prueba Shapiro- Wilk: p> ,05), mientras que la prueba no paramétrica U de Mann- Whitney, se utilizó para las variables con presencia de asimetría y en donde se rechazó la prueba de hipótesis de normalidad (IMC y puntaje total PSQI, prueba Shapiro- Wilk: p> ,05).

Se analizó por separado la asociación entre cada variable del sueño (calidad del sueño y cantidad de horas de sueño por noche) con el IMC en forma continua a través de dos modelos de regresión logística ajustados por edad, sexo y ocupación. Se seleccionaron estas variables de ajuste ya que se vinculan estrechamente con el sueño y el peso corporal en los adolescentes, siendo potenciales variables de confusión^9^^,^^23^. En los modelos ajustados no se rechazó la prueba de bondad de ajuste (prueba Hosmer- Lemeshow, p> ,05).

Asimismo, se exploraron por separado dos modelos de regresión lineal tomando en cuenta como variable desenlace el IMC y como exposición, para el modelo 1, la calidad de sueño (PSQI> 5 puntos y PSQI< 5 puntos) y la cantidad de horas de sueño por noche (> 6 horas y < 6 horas) para el modelo 2. Ambos modelos fueron ajustados por las variables de confusión anteriormente mencionadas (edad, sexo y ocupación). Se utilizó la transformación logarítmica del IMC porque se rechazó la hipótesis nula de normalidad (prueba sesgo- curtosis D'Agostino, p< ,05). Los modelos de regresión lineal ajustados cumplieron los supuestos de normalidad, linealidad y homocedasticidad (p> ,05).

Se utilizó el software estadístico Stata 14.0 para Windows (StataCorp LLC, College Station, TX, EE. UU), considerándose diferencias estadísticamente significativas si p< ,05 y marginales si ,05 < p < ,10, además de reportarse intervalos de confianza (IC) del 95%. La base de datos fue almacenada en Mendeley Data^24^.

### Consideraciones Éticas

El protocolo del estudio fue aprobado por el Comité de Ética y de Investigación de la FET- UAT (No. Oficio: 003-2018), considerándose los lineamientos éticos de la Declaración de Helsinki^25^ y la Ley General de Salud en materia de Investigación en Salud^26^. Los estudiantes brindaron su autorización para participar en el estudio al firmar el Consentimiento Informado previo a la colecta de la información.

## Resultados

La edad promedio de los estudiantes fue de 18,21 años (DE= ,42 años), 82,84% fueron mujeres, 92,54% estudiantes de dedicación exclusiva y 99,25% en estado civil de soltería. La mediana del IMC fue de 24,27 kg/m2 (Q1 - Q3= 21.29 - 28,16 kg/m2), la prevalencia de sobrepeso u obesidad fue de 45,52% (IC 95%= 35,46 - 52,86%). El puntaje promedio del PSQI fue de 5,41 puntos (DE= 2,28 puntos). El promedio de la cantidad de horas de sueño por noche fue de 6,68 horas (DE= 1,06 horas), un 44,03% reportó dormir < 6 horas por noche, y uno de cada dos participantes refirió una mala calidad de sueño en las últimas cuatro semanas. No se observaron diferencias en el puntaje total del PSQI (p= ,51) o el promedio de la cantidad de horas de sueño por noche (p= ,89) entre hombres y mujeres.

Las Tablas 1 y 2 muestran las características sociodemográficas y de salud por grupos de calidad y cantidad de sueño. En comparación con el grupo con una buena calidad de sueño, en el grupo con una mala calidad de sueño, quienes obtuvieron una mediana del puntaje en el PSQI de 7 puntos (Q1 - Q3= 6 - 8 puntos), se observó una mayor cantidad de participantes dedicados exclusivamente al estudio (p= .04), así como un menor promedio de la cantidad de horas de sueño por noche (p < ,01). En el grupo con una duración de sueño < 6 horas (5,64 horas, DE= ,48 horas), se observó una tendencia en el promedio del IMC (p= ,07) y una mediana superior del puntaje total del PSQI (p < ,01) en comparación con aquellos que reportaron dormir la cantidad de horas recomendada. En general, no se observaron otras diferencias en las variables restantes entre los grupos.

**Tabla 1:** Características sociodemográficas y de salud de los participantes por calidad y cantidad de sueño

Característica	Buena calidad de sueño, PSQI < 5 n= 67	Mala calidad de sueño, PSQI >5 n= 67	Valor-p	Cantidad de horas de sueño >6h n= 75	Cantidad de horas de sueño <6h n= 59	Valor-
Edad, años	18,22 (,41)	18,19 (,39)	,80	18,20 (,40)	18,22 (,41)	,66
IMC, kg/m2	24,63 (21,30 - 28,16)	24,27 (21,09 - 28,19)	,95	24,49 (4,74)	26,12 (5,64)	,07
Puntaje total PSQI	4 (3 - 5)	7 (6 - 8)	< ,01	4 (3 - 6)	6 (5 - 8)	< ,01
Cantidad de sueño, h	7,16 (,94)	6,20 (,92)	< ,01	7,50 (,55)	5,64 (,48)	< ,01

*Valor-p de pruebas t- Student o U- Mann- Whitney. Se reporta media (DE) o mediana (Q1 - Q3) n= 134*

**Tabla 2:** Características sociodemográficas y de salud de los participantes por calidad y cantidad de sueño (cont.)

Característica	Buena calidad de sueño, PSQI < 5 n= 67		Mala calidad de sueño, PSQI >5 n= 67		Valor-p	Cantidad de horas de sueño >6h n= 75		Cantidad de horas de sueño <6h n= 59		Valor-p
	n	%	n	%		n	%	n	%	
Sexo					,81					,32
Hombre	12	17,91	11	16,42		15	20,00	8	13,56	
Mujer	55	82,09	56	83,58		60	80,00	51	86,44	
Ocupación					,04					,18
Estudiante	59	88,06	65	97,01		67	89,33	57	96,61	
Estudiante y trabajador	8	11,94	2	2,99		8	10,67	2	3,39	
Estado Civil					1,00					1,00
Soltero	66	98,51	67	100,00		74	98,67	59	100,00	
Casado	1	1,49	0	0,00		1	1,33	0	0,00	
IMC					,48					,56
Bajo peso	7	10,45	3	4,48		6	8,00	4	6,78	
Peso normal	29	43,28	34	50,75		38	50,67	25	42,37	
Sobrepeso	16	23,88	18	26,87		19	25,33	15	25,42	
Obesidad	15	22,39	12	17,91		12	16,00	15	25,42	

*Valor-p de pruebas Ji- Cuadrada de independencia o exacta de Fisher n= 134*

Los resultados de los modelos de regresión logística ajustados por edad, sexo y ocupación se muestran en la Tabla 3. No se observó una asociación entre la calidad del sueño y el IMC (modelo 1, OR= 1,01; IC 95%= ,94 - 1,08; z= ,31; p= ,75). Sin embargo, el incremento en el IMC se asoció con una mayor probabilidad de dormir 6 horas o menos (modelo 2, OR= 1,08; IC 95%= 1,01 - 1,16; z= 2,25; p= ,02).

**Tabla 3:** Regresión logística de la probabilidad de una mala calidad y cantidad de sueño por IMC

Modelo 1* Mala calidad de sueño vs Buena calidad de sueño	OR	IC 95%		z	Valor- p
Índice de Masa Corporal, kg/m2	1,01	,94	1,08	,31	,75
Sexo, mujer	,98	,37	2,57	-,04	,97
Edad, años	1,03	,44	2,39	,08	,93
Ocupación, estudia y trabaja	,21	,04	1,10	-1,84	,06
Modelo 2*	OR	IC 95%		z	Valor- p
*<6* h de sueño vs >6 h de sueño					
Índice de Masa Corporal, kg/m2	1,08	1,01	1,16	2,25	,02
Sexo, mujer	1,95	0,70	5,42	1,29	,19
Edad, años	1,53	0,64	3,64	,97	,33
Ocupación, estudia y trabaja	,25	0,04	1,32	-1,63	,10

*Variables de ajuste: sexo, edad y ocupación n= 134

La Tabla 4 muestra los resultados de los modelos de regresión lineal ajustados por edad, sexo y ocupación. De forma general, las conclusiones se mantienen al no observarse una asociación entre la calidad del sueño y el IMC (modelo 1, p= ,01; IC 95%= -,05, ,08; p= ,71); mientras que la cantidad de horas de sueño por noche se asoció negativamente con el IMC, los estudiantes que reportaron dormir > 6 horas por noche tuvieron en promedio 2,03 kg/m2 menos de IMC en comparación con aquellos que no cumplieron las horas recomendadas de sueño (modelo 2, p= -,07; IC 95%= -,14, ,00; p= ,02).

**Tabla 4:** Regresión lineal de la calidad y cantidad de sueño con el IMC^1^

Modelo 1* Índice de Masa Corporal (kg/m2)	P	IC 95%		t	Valor- p
Calidad del sueño, PSQI > 5 puntos a	,01	-,05	,08	,36	,71
Sexo, mujer	-,11	-,20	-,02	-2,46	,01
Edad, años	-,05	-,13	,02	-1,29	,19
Ocupación, estudia y trabaja	,06	-,06	,20	1,00	,31
Modelo 2* Índice de Masa Corporal (kg/m2)	P	IC 95%		t	Valor- p
Cantidad de sueño, >6 horas b	-,07	-,14	,00	-2,22	,02
Sexo, mujer	-,12	-,21	-,03	-2,65	< ,01
Edad, años	-,05	-,14	,02	-1,46	,14
Ocupación, estudia y trabaja	,08	-,04	,21	1,26	,21

f *Logaritmo del IMC n= 134*

*Variables de ajuste: sexo, edad y ocupación

a *El grupo de referencia fue PSQI < 5 puntos*

b *El grupo de referencia fue < 6 horas*

## Discusión

En este estudio, no se observó una asociación entre la calidad del sueño y el Índice de Masa Corporal entre los estudiantes universitarios, mientras que el incremento en el Índice de Masa Corporal se asoció con una mayor probabilidad de dormir 6 horas o menos, después de considerar otras variables como la edad, el sexo y la ocupación. Conclusiones que se mantienen cuando se analizó la asociación de estas variables en forma lineal, donde la cantidad de horas de sueño por noche se asoció negativamente con el Índice de Masa Corporal, asociación que no se observó con la calidad de sueño. Estos resultados aportan evidencia en una asociación que no ha sido del todo clarificada en adolescentes universitarios, a pesar de esto, la interpretación debe realizarse con cautela debido al diseño del estudio.

Los hallazgos coinciden con diversos autores, quienes han reportado asociaciones negativas entre la cantidad de horas de sueño con el Índice de Masa Corporal. En una población de adultos italianos (> 29 años), el promedio del IMC se redujo entre 2-3 kg/m2 por cada incremento en una hora de sueño^7^; asociación que se observó en menor magnitud en otros estudios, por ejemplo, en población de estudiantes universitarios de Psicología en Estados Unidos, se redujo el promedio del IMC en 0,38 kg/m2 por cada incremento en el puntaje del factor de duración de sueño del Índice de Calidad de Sueño de Pittsburgh^12^. En estudiantes de una universidad en China, el IMC predijo la duración del sueño, de tal forma que por cada kg/m2 que se incrementó el IMC, se observó una reducción promedio de 22 minutos en la cantidad total de sueño^17^. En adolescentes coreanos, por cada hora que se redujo la cantidad promedio de sueño, se observó un incremento de 0,15 kg/m2 en el IMC^15^. Finalmente, en adultos europeos (18- 39 años), por cada hora que aumentó la cantidad de sueño, el IMC disminuyó en 0,086 kg/m2 ^23^.

Se encontraron diferencias en la asociación con lo reportado por Hayes et al. referente a la duración del sueño con el IMC, en donde no se observó un vínculo entre estas variables. Sin embargo, se coincide con estos autores y con Pilcher et al. en la asociación entre la calidad de sueño con el IMC, al no encontrar una asociación significativa^9^^,^^12^. El primer estudio se realizó en adolescentes australianos participantes de un proyecto para la reducción de peso, mientras que el segundo fue llevado a cabo en población americana universitaria. Este hallazgo no queda dilucidado en su totalidad, ya que recientemente se ha reportado que una mala calidad de sueño se asocia con una mayor probabilidad de padecer sobrepeso u obesidad^10^, por lo que se insta a continuar analizando este vínculo utilizando diseños de estudios donde se considere el seguimiento de los participantes.

Es posible que las diferencias observadas fueran debido a la población estudiada, los instrumentos utilizados para medir la calidad y la cantidad de sueño, así como el número y el tipo de variables de ajuste consideradas en la estimación de los coeficientes. Por ejemplo, en algunos estudios, al ajustar por la actividad física o la calidad de la alimentación, las asociaciones tendieron a reducirse considerablemente^12^^,^^17^^,^^23^.

Dentro de los diversos mecanismos que explican la asociación entre la cantidad de horas de sueño por noche con el incremento del Índice de Masa Corporal, se encuentran las alteraciones hormonales, los factores conductuales y el entorno. Una duración de sueño reducida afecta los niveles de ciertas hormonas que alteran el control del apetito como la leptina, la grelina y la melatonina. La primera de ellas es producida principalmente por los adipocitos, cumpliendo la función de suprimir el apetito; la segunda, es liberada por el estómago y se encarga de estimular el apetito. Se ha reportado una reducción del 18% en el promedio de los niveles de leptina y un incremento promedio del 28% en los niveles de grelina durante periodos de falta de sueño (< 4 horas durante 6 días) a causa de la mayor demanda energética producto de la vigilia prolongada^5^^,^^10^. Además, como resultado de la exposición nocturna a la luz por la vigilia, se reducen drásticamente los niveles de melatonina, que interviene en el crecimiento y la actividad del tejido adiposo, favoreciendo el incremento de las reservas de adipocitos blancos^8^.

Los factores conductuales afectan en la cantidad y la calidad de alimentos que ingieren las personas, así como en la práctica de actividad física. Las personas que duermen una cantidad insuficiente de horas son más propensas a consumir alimentos hipercalóricos y muestran una menor adherencia a la dieta mediterránea, la cual se asocia inversamente con la obesidad^7^. Inclusive, se ha hipotetizado que este consumo hipercalórico se deriva de una conducta hedónica, puesto que con la disminución en la cantidad de sueño se mejora la actividad cerebral en las zonas de recompensa^27^, o también de conductas que promueven la ingesta de aperitivos, como el pasar mayor tiempo frente a pantallas y el uso de Internet^9^. Aunado a ello, las personas con una cantidad de sueño deficiente refieren fatiga y somnolencia diurna, que disminuye su motivación para realizar actividad física^8^.

Asimismo, los cambios en el entorno socioeconómico, que provoca el incremento en las exigencias académicas; los programas en los cuales se trabaja por turnos, como el caso de los de ciencias de la salud, en donde se busca el desarrollo paralelo de la teoría y la práctica; y el tiempo prolongado de desplazamiento entre el hogar y las instituciones, son algunos factores del entorno que afectan en la calidad y la cantidad de sueño^5^^,^^17^. De esta forma, la acumulación de tejido adiposo, en conjunto con el incremento del apetito basado en la ingesta hipercalórica derivado de la elevación en los niveles de grelina y los factores conductuales o del entorno, provoca inevitablemente un aumento en el peso corporal.

El presente estudio tiene varias limitaciones. Primeramente, el diseño transversal dificulta el establecimiento de una asociación causal; segundo, el muestreo por conveniencia posibilita la presencia de sesgo de selección y, por último, la presencia de confusión residual. Este sesgo se presenta cuando no se controlan adecuadamente en el diseño o el análisis del estudio algunas variables que se asocian con la exposición y con el desenlace de forma causal, lo que resulta en una sobre o subestimación de la asociación real. En el presente, la falta de medición de otras variables vinculadas directamente con el sueño y el IMC (como los hábitos alimenticios y la actividad física) afectaría en las estimaciones de los coeficientes ajustados^28^.

Los hallazgos conllevan ciertas implicaciones en el ámbito académico, al respecto, diversos autores han propuesto una serie de estrategias educativas enfocadas en la modificación de creencias erróneas en torno al sueño (como pensar que una cantidad insuficiente de sueño no produce consecuencias severas a largo plazo o considerar que las horas perdidas de sueño se pueden recuperar), y la educación en salud para mejorar la higiene del sueño, brindar asesoría para modificar los patrones de sueño de las personas, evitar el consumo de sustancias (nicotina, cafeína y alcohol) y la realización de ciertas actividades (tiempo frente a pantallas y actividad física vigorosa) que alteran el patrón del sueño previo a la hora de acostarse^8^, así como la reducción en los niveles de estrés, debido a que éste afecta negativamente el peso corporal, la duración y la calidad del sueño^29^^,^^30^. Estrategias que pueden ser implementadas en los programas educativos de las instituciones de educación superior.

## Conclusiones

La cantidad de horas de sueño se asoció negativamente con el Índice de Masa Corporal en adolescentes universitarios mexicanos, no se observó una asociación entre la calidad de sueño y el Índice de Masa Corporal. Se recomienda la realización de estudios de corte longitudinal en poblaciones similares considerando un muestreo probabilístico, con la finalidad de profundizar en la comprensión de la asociación causal entre estas variables; además de la inclusión de otros factores que se asocien tanto con el sueño como con el Índice de Masa Corporal, reduciendo potenciales sesgos en las estimaciones.
